# Supplementary material for: Parrotfish corallivory on stress-tolerant corals in the Anthropocene
Source: PLoS One. 2021 Sep 9;16(9):e0250725. doi: 10.1371/journal.pone.0250725 (PMC8428567; doi:10.1371/journal.pone.0250725)
Supplement: S1 File — (DOCX) [file pone.0250725.s001.docx]

**ELECTRONIC SUPPLEMENTAL MATERIAL**

**Supplemental methods**

*Parrotfish and benthic surveys*

To avoid unconscious bias when surveying parrotfish abundance and estimating coral cover, surveyors in 2018 (VH and RM) did not access the data from 2008.

*Mapping reef zones*

To provide a reef-wide estimate of parrotfish corallivory at the two time periods, we calculated the weighted average number of scars across the study location by mapping the four reef zones (slope, crest, flat, and back reef) using the software Google Earth Pro. The reef slope was identified visually by a darker blue colour due to deeper water. The light, brown-colored crest was identified as a narrow strip on the exposed side of the reef lining the reef edge adjacent to the slope. The reef flat was identified by a uniform turquoise area behind the crest that was mostly devoid of topographic features. Finally, the back reef was located behind the flat and identified by a darker shade of mottled blue and a noticeable increase in patchiness of the reef area adjacent to the lagoon.

**Supplemental tables**

Table S1. Performance of generalized linear models (GLMs) with a negative binomial distribution where parrotfish abundance is the response variable and ‘Year’ (2008, 2018) and ‘Zone’ (slope, crest, flat, back) are included as fixed effects. df: degrees of freedom; LL: maximum loglikelihood of the model; AICc: corrected Akaike Information Criterion; ∆AIC: delta Akaike Information Criterion; wAIC: Akaike Information Criterion weights. wAIC value of 1 indicates 100% confidence that the model is the most parsimonious model for inference, and 0 indicates 0% confidence. Most parsimonious model is shown in bold.

| **Model** | **df** | **LL** | **AICc** | **∆AIC** | **wAIC** |
| --- | --- | --- | --- | --- | --- |
| **Parrotfish abundance ~ Year x Zone** | **9** | **-430.123** | **879.9** | **0** | **1** |
| Parrotfish abundance ~ Year + Zone | 6 | -443.143 | 899.0 | 19.14 | 0 |
| Parrotfish abundance ~ Zone | 5 | -445.489 | 901.5 | 21.61 | 0 |
| Parrotfish abundance ~ Year | 3 | -451.830 | 909.9 | 29.97 | 0 |
| Parrotfish abundance | 2 | -455.977 | 916.1 | 36.16 | 0 |

Table S2. Tukey’s pairwise post-hoc comparisons of parrotfish abundance between surveys conducted in 2008 and 2018 across the four reef zones at Lizard Island. Tests were performed in the log scale. SE: Standard Error of the mean; ns: Non-significant; *: Significant.

| **2018 vs 2008** | **Ratio** | **SE** | **Z ratio** | **P value** | **Significance** |
| --- | --- | --- | --- | --- | --- |
| Slope | 1.633 | 0.431 | 1.860 | 0.063 | ns |
| Crest | 1.471 | 0.375 | 1.513 | 0.130 | ns |
| Flat | 3.499 | 0.892 | 4.911 | < 0.001 | * |
| Back | 0.496 | 0.128 | -2.716 | 0.007 | * |

Table S3. Parrotfish predation scars on massive *Porites* in 2008 and 2018 at the study site, Lizard Island, on the Great Barrier Reef. SE: Standard Error of the mean.

|  | **Mean scars m^-2^ ± SE** | |
| --- | --- | --- |
| **Zone** | **2008** | **2018** |
| Slope | 57.5 ± 3.8 | 141.4 ± 25.7 |
| Crest | 670.7 ± 80.8 | 278.1 ± 51.1 |
| Flat | 735.4 ± 129.3 | 182.4 ± 50.5 |
| Back | 58.8 ± 6.9 | 86.5 ± 14.9 |

Table S4. Summary of the generalized linear model results used to evaluate the probability of a massive *Porites* colony being bitten at Lizard Island, on the Great Barrier Reef. Habitat, colony area, and their interaction are included as fixed effects. SE: Standard Error of the mean; ns: Non-significant.

| **Coefficients** | **Estimate** | **SE** | **Z value** | **P value** | **Significance** |
| --- | --- | --- | --- | --- | --- |
| (intercept) | -0.044 | 0.144 | -0.305 | 0.760 | ns |
| Crest | 0.043 | 0.287 | 0.149 | 0.882 | ns |
| Flat | -0.273 | 0.294 | -0.930 | 0.352 | ns |
| Back | 0.341 | 0.220 | 1.549 | 0.121 | ns |
| Colony area | 0.593 | 0.944 | 0.629 | 0.529 | ns |
| Crest * colony area | 7.657 | 6.382 | 1.200 | 0.230 | ns |
| Flat * colony area | 0.642 | 2.634 | 0.244 | 0.807 | ns |
| Back * colony area | -0.037 | 1.058 | -0.035 | 0.972 | ns |

Table S5. Summary of the generalized linear model results used to evaluate the relationship between the density of scars and the area of massive *Porites* colonies at four reef zones at Lizard Island, on the Great Barrier Reef. Habitat, log10(colony area), and their interaction are included as fixed effects. SE: Standard Error of the mean; ns: Non-significant; *: Significant.

| **Coefficients** | **Estimate** | **SE** | **Z value** | **P value** | **Significance** |
| --- | --- | --- | --- | --- | --- |
| (intercept) | 2.848 | 0.248 | 11.507 | <0.001 | * |
| Crest | 0.926 | 0.501 | 1.849 | 0.064 | ns |
| Flat | -0.619 | 0.533 | -1.161 | 0.246 | ns |
| Back | 1.035 | 0.291 | 3.554 | <0.001 | * |
| log10(colony area) | -1.664 | 0.163 | -10.219 | <0.001 | * |
| Crest * log10(colony area) | 0.379 | 0.292 | 1.299 | 0.194 | ns |
| Flat * log10(colony area) | -0.597 | 0.355 | -1.682 | 0.093 | ns |
| Back * log10(colony area) | 0.741 | 0.208 | 3.573 | <0.001 | * |

**Supplemental figure**

**Fig S1.** **Density plots showing the size distribution of massive *Porites* corals across the study location in 2018 at Lizard Island, on the Great Barrier Reef.** Colony size was estimated based on the planar surface area. Data is presented in the log scale.
