# Supplementary material for: Parrotfish corallivory on stress-tolerant corals in the Anthropocene
Source: PLoS One. 2021 Sep 9;16(9):e0250725. doi: 10.1371/journal.pone.0250725 (PMC8428567; doi:10.1371/journal.pone.0250725)
Supplement: Attachment — Submitted filename: Response to Reviewers.docx [file pone.0250725.s002.docx]

**Response to Reviewers**

*“Reduced parrotfish corallivory on stress-tolerant corals in the Anthropocene”*

1. Please ensure that your manuscript meets PLOS ONE's style requirements, including those for file naming. The PLOS ONE style templates can be found at:

<https://journals.plos.org/plosone/s/file?id=wjVg/PLOSOne_formatting_sample_main_body.pdf> and

<https://journals.plos.org/plosone/s/file?id=ba62/PLOSOne_formatting_sample_title_authors_affiliations.pdf>

*Thank you. We have made sure that the manuscript meets PLOS ONE’s style requirements.*

2. In your Methods section, please provide additional location information of the study location, including geographic coordinates for the data set if available.

*Lines 101/102 - We have inserted the GPS coordinates of the study location.*

3. In your Methods section, please provide additional information regarding the permits you obtained for the work. Please ensure you have included the full name of the authority that approved the field site access and, if no permits were required, a brief statement explaining why.

*Lines 129/131 – as requested, we have added a statement including the permits obtained to carry out this study.*

4. We note that you have stated that you will provide repository information for your data at acceptance. Should your manuscript be accepted for publication, we will hold it until you provide the relevant accession numbers or DOIs necessary to access your data. If you wish to make changes to your Data Availability statement, please describe these changes in your cover letter and we will update your Data Availability statement to reflect the information you provide.

*Thank you. We will upload the data to the James Cook University data repository and we will provide the relevant accession numbers once the process has been completed and these numbers become available.*

5. We note that Figure 1b in your submission contains satellite images which may be copyrighted.

All PLOS content is published under the Creative Commons Attribution License (CC BY 4.0), which means that the manuscript, images, and Supporting Information files will be freely available online, and any third party is permitted to access, download, copy, distribute, and use these materials in any way, even commercially, with proper attribution. For these reasons, we cannot publish previously copyrighted maps or satellite images created using proprietary data, such as Google software (Google Maps, Street View, and Earth). For more information, see our copyright guidelines: <http://journals.plos.org/plosone/s/licenses-and-copyright>.

We require you to either (a) present written permission from the copyright holder to publish this figure specifically under the CC BY 4.0 license, or (b) remove the figure from your submission:

a. You may seek permission from the original copyright holder of Figure 1b to publish the content specifically under the CC BY 4.0 license.

We recommend that you contact the original copyright holder with the Content Permission Form ([http://journals.plos.org/plosone/s/file?id=7c09/content-permission-form.pdf](about:blank)) and the following text:

“I request permission for the open-access journal PLOS ONE to publish XXX under the Creative Commons Attribution License (CCAL) CC BY 4.0 ([http://creativecommons.org/licenses/by/4.0/](about:blank)). Please be aware that this license allows unrestricted use and distribution, even commercially, by third parties. Please reply and provide explicit written permission to publish XXX under a CC BY license and complete the attached form.”

Please upload the completed Content Permission Form or other proof of granted permissions as an "Other" file with your submission.

In the figure caption of the copyrighted figure, please include the following text: “Reprinted from [ref] under a CC BY license, with permission from [name of publisher], original copyright [original copyright year].”

*We have replaced the satellite image with a photograph of the study site taken from a plane by the lead author. Both photographs on Figure 1 are therefore taken by the lead author of the manuscript. We have noted this in the figure legend (Line 118).*

**Additional Editor Comments:**

I have reviewed the manuscript in addition to the external reviewer and I agree with the external reviewer that there are a number of issues that will need to be addressed before the manuscript can be reconsidered for publication. Reviewer 1 makes some important points about the potential for bias in the way bites were measured between different size colonies and these issues need to be addressed to ensure readers that there are unlikely to be artefacts in how measurements were taken.

I also agree that the analyses need to be consistent and if there is disagreement with reviewer 1's comments then a clear explanation should be given.

I would like to see some more detail about what parrotfish species are scraping the porites colonies - is it all species or just a small group? If a small group then should you have concentrated on counts of those species in your analysis.

*It is not possible to identify species-specific scars. Almost all of the scars were made by parrotfishes of the genus Scarus (these can be separated from Chlorurus), however, species known to bite on live corals in the GBR are now listed (with references supporting this list) in the main text:*

*Lines 80/83 – “On the Great Barrier Reef, multiple parrotfish species have been reported to bite on massive Porites, including Scarus flavipectoralis, Scarus niger, Scarus frenatus, Scarus rivulatus, Chlorurus microrhinos, Chlorurus spilurus, Cetoscarus ocellatus, and Bolbometopon muricatum* [1–3]*”. (Bellwood 1985, Bellwood and Choat 1990, Bonaldo and Bellwood 2011)*

While I found the manuscript to be well written my overall assessment is that the arguments presented within the manuscript are not really supported by the data and there are other explanations for results that are not equally explored. This is an observational study and there needs to be other supporting work to back up the idea that smaller colonies are being preferentially targeted. Moreover the authors fail to explore other reasons for the decline in overall feeding scars on porites. There are too many opinions inserted in the manuscript and overuse of emotive statements.

*We have modified the text to be less speculative where possible and put forward hypothesis that could be tested in the future - which could help explain the patterns we documented.*

The argument in the manuscript is not consistent as it oscillates between stating that massive porites are subject to less predation by parrotfish in a disturbed environment but that predation may be adversely affecting recruitment and rate of transitions of juvenile to adult porites.

*Thank you. This needs to be clear. Our results showed that overall predation pressure appears to have diminished substantially at the study location. However, they also showed that scar densities were higher on smaller colonies, which suggests that earlier life stages are particularly vulnerable to predation. As the editor points out, this is typical of other coral populations. Both phenomena are compatible and reveal an interesting pattern: while overall coral predation declined on the study location following mass coral mortality, the comparatively higher predation pressure we documented on smaller colonies may represent a sustained energetic burden to young corals. This highlights that reduced overall parrotfish corallivory on massive Porites may not necessarily release massive Porites populations from the adverse effects of predation. The main text has been modified to clarify this issue (lines 482/486).*

line 72-73 What species are the main scrapers on porites corals - lumping all parrotfish together is not a powerful test

*Our data relied on the marks left by parrotfish feeding on massive Porites. Therefore, it is not possible to tease apart which parrotfish species produced the scar. We agree with the editor that this is an interesting question worth addressing in the future, but this will require a different methodology that involves direct feeding observations.*

line 82-83 This statement has no supporting evidence and should be removed.

*Lines 94/95 - We have edited this statement to refer specifically to reefs around Lizard Island. We cite two papers which have documented a major loss of coral cover that has resulted in changes in coral communities. Thank you for bringing this to our attention.*

line 87 most corals have been lost is not a precise statement - do you mean they have gone extinct or severely reduced in abundance?

*Sorry for the lack of clarity. This sentence refers to the reduction in the abundance, not to extinctions. We have clarified this in the text, which now reads as follows:*

*Line 100 - …while most corals, except Porites, have become less abundant (especially acroporid corals).*

line 92 don’t need to add "stress-tolerant corals" your hypothesis is has reef degradation affected the intensity of parrotfish predation

*Line 104 – changed as suggested.*

line 94-95 "parrotfish are a growing threat to the persistence of corals on the GBR" is completely unsupported and this type of highly emotive statement has no place in the manuscript

*Line 105 - We have removed this sentence as suggested.*

line 101 - replace good with appropriate

*Line 111 - Changed as suggested.*

line 124-125 this sentence is unclear - do you mean that photos were taken from a consistent distance above the substrate?

*Line 160 - Yes. Images were obtained at a distance from the benthos of approximately 1.5 m. We have clarified this in the main text.*

line 127 how did you decide on the number of points to analyse?

*Line 150 - We followed the methodology used by Bonaldo and Bellwood (2011) to ensure consistency between our dataset and theirs. We have included this in the main text.*

lines 140-146 this methodology needs to be more clearly explained or addressed as reviewer #1 has pointed out.

*Thank you. We have addressed this point in reviewer #1’s comments below.*

lines 148 - 175 statistical tests need to be addressed as per reviewer 1's comments

*We have addressed this issue in reviewer #1’s comments below.*

line 234 You appear to be extrapolating way outside your study area. You have no evidence to support doing this. Given the degrees of patchiness that exist on reefs how do you know that the patterns observed in your study area are uniformly repeated across the entire reef

*We note the editor’s concerns and we have modified the text accordingly to acknowledge that this comparison does not extend beyond our study site and assumes a uniform distribution of massive Porites. In addition, we have moved this text to the discussion section. The text now reads as follows:*

*Lines 347/359 – “The apparent reduction in predation pressure is best illustrated by the difference in the overall density of scars. Considering, for example, a typical section of reef from the study site (composed of 35.2% reef flat, 27.3% of back reef, 24.2% of reef slope, and 13.3% of crest; as measured from satellite images, ESM Methods), using the Porites cover (Table 1 and Fig 1, respectively) and assuming a uniform distribution of Porites colonies yields a ~40% reduction in the mean density of scars at the study location from 2008 to 2018 (from 676 to 400 scars per 100 m^2^ of massive Porites planar surface area).”*

lines 293-299 You do not explore the equally plausible reason for decreased predation on porites is because parrotfish have found plenty of food around other than porites. The changes don’t have to be manifest as increased numbers they can equally be a shift in feeding behaviour.

*Thanks for pointing this out. We agree that it is worthwhile expanding on this hypothesis to include a potential shift in the foraging behaviour of parrotfishes. We have incorporated it into the main text as a new paragraph. The text reads as follows:*

*Lines 375/385 - Most parrotfishes target turf algae-covered surfaces on the reef* [4]*. Thus, the observed overall reduction in scars on massive Porites may reflect a shift in the foraging behaviour of parrotfishes driven by an increase in the availability of turf-covered substratum following mass coral mortality* [5]*. While we cannot confirm that this was the case at Lizard Island, changes in the foraging behaviour of parrotfishes following coral mortality have been documented at other locations* [6]*.*

line 305 saying that predation moved in an unexpected direction is your opinion. As I have stated above there is another opinion that the change you saw was not unexpected. You need to stick to supported facts or else note that there are numerous possibilities none of which you have made a convincing case for.

*Thank you. We have edited the text to note that the decrease in number of scars could also be due to a shift in the foraging behaviour of parrotfishes (line 362) and we have elaborated on this hypothesis in lines 375/385.*

lines 312-314 Why do you think predation was highest on the crest and reef flat. There is plenty of literature to support this pattern such as increased algae productivity?

*We have inserted the following text in the discussion to lay out the potential reasons for higher parrotfish corallivory in the crest and reef flat:*

*Lines 400/402 – On a mid-shelf reef such as Lizard Island, these shallow reef habitats typically concentrate the largest foraging activity of parrotfishes regardless of whether they graze on turf algae surfaces* [7] *or predate on live coral* [1]*.*

line 318 "as one may expect" you must stop inserting your opinion as fact into the manuscript. just state the neutral case i.e. that you found scar numbers increased with colony size

*Line 416 - We have removed this text from the sentence. The text now reads “The number of parrotfish scars on massive Porites increased with colony size.”.*

lines 325-326 This would seem to be an avenue for further investigation. If there is targeted feeding of smaller colonies then all the characteristics of feeding on younger corals should be investigated. What about nutritional values?

*This is a very interesting point. Smaller colonies may be preferred targets for parrotfishes because they are easier to bite since their surface is more concave. Or perhaps they have higher nutritional value, thicker tissues, etc. We hope that our findings lead to these exciting future studies.*

lines 333-338 Juveniles of most species will experience higher mortality rates than adults this is not confined to porites corals. You would have to demonstrate that recruitment rates and juvenile abundance/densities declined below long-term rates to be able to support this statement.

*We agree with the editor that lower survival of juveniles is a common life-history trait of all corals and we have included a statement in the main text to acknowledge this. We also note that massive Porites (and other massive corals) have been found to be more vulnerable to severe weather when they are young (contrary to habitat-forming corals such as Acroporas which become more vulnerable as they grow). Since our study location was swept by two back-to-back cyclones, we propose in the discussion that this could have resulted in a higher-than-expected mortality of small massive Porites. Since we documented higher scar densities on small colonies, a decline in young Porites could have contributed to the overall reduction in observed scar density between the two time periods. We have now inserted the following new text in the discussion:*

*Lines 432/443 - This is particularly relevant if we consider the patterns of coral mortality associated with tropical cyclones. Lower survivorship of juveniles is a generalised feature of corals – and indeed all other animals. In corals, structurally-complex morphotypes (e.g., tabular, corymbose corals) become increasingly more vulnerable to hydrodynamic forces as they grow in size* [8]*. However, massive corals are disproportionally impacted by intense wave action (such as those generated by cyclones Ita and Nathan) when they are small* [9]*. Thus, it is likely that the abundance of juvenile massive Porites decreased over the study period due to differential mortality of juveniles following the impact of cyclones Ita, and especially Nathan. This could have contributed to the decline in parrotfish scars.*

lines 355-356 You have provided no evidence that there has been a decline in juvenile corals through predation by parrotfish.

*Indeed, we cannot state that there has been a decline in juvenile massive Porites due to parrotfish corallivory. However, we provide evidence of higher scar densities on small colonies and we discuss the possibility that this may contribute to colony mortality at this vulnerable stage.*

lines 370-371 But you have not made the case that the level of predation on juvenile porites is any worse than previously. In fact it could be argued that access to increased algal/detrital resources as a result of disturbance has decreased the impacts of feeding scars as parrotfish have many other feeding opportunities

*Indeed, this is a possibility. We now see that this statement was not clear. We have edited this statement to incorporate this caveat and to increase clarity. The text now reads as follows:*

*Lines 482/486 – “Although colony-size specific parrotfish predation on massive Porites has not been documented on undisturbed reefs, if the observed pattern of disproportionately higher scar density on small colonies is a persistent pattern, parrotfish corallivory on Indo-Pacific reefs could represent an important factor modulating the dynamics of remaining massive Porites populations in the Anthropocene.”*

**Review Comments to the Author**

Reviewer #1: This manuscript studied patterns of coral communities and feeding by coral-eating fishes (parrotfishes) in 2018 and compared these with the data in 2008. The comparison of these results between 2008 and 2018 was well described and the supporting data and analyses were fine. However, I am concern about the method and analysis used to determine Porites size and bites by parrotfishes and might include possible artefacts due to the survey methodology, as discussed below. Also, statistical analysis should be consistent, Information theory and Frequentist particularly should not be mixed in a single analysis for the same purpose.

*Thank you for reviewing our manuscript. We have addressed each comment below on a point-by-point basis.*

In the photoquadrat survey, how the photos are taken and analysed should be more cleared. Looking at the large Porites colony in Fig. 4, it appears that the sides (which more likely to be bitten?) and the shadows of the large knots, were not being photographed. The lower density of bites on the larger Porites could simply be due to the increased blind spot of the camera as the size of the colony increases, so that many of the bites are not captured. In short, “surface area” of Porites in this manuscript seems to be more correctly “projected area”.

*We considered this issue carefully when designing the study. However, to ensure consistency across images, all photos were taken from above capturing the colony’s planar surface area, a term that we chose because it is commonly used to refer to the visible surface of a coral colony viewed from above. To increase clarity, we now refer to it as “horizontal planar surface area” (line 178).*

*We are not aware of any study showing that parrotfishes show preference for biting on the sides (or the top) of massive Porites colonies. Although this cannot entirely be ruled out, we observed scars present all around colonies with no discernible preference for a particular location of colonies.*

*We acknowledge that because we photographed colonies from above scars inflicted on the sides of colonies could not be included in our counts. Our estimates of scar counts are conservative (i.e., underestimate total scars) because we followed a standard methodology across all size classes so that the number of scars could be compared per unit area avoiding a sampling bias. As suggested by the reviewer, we now explicitly state that:*

*Lines 179/181 - “All images were taken from the same position (i.e., directly above the colony) to ensure consistency across all colonies sampled”.*

I wonder that the ratio of diameter to height changes with growth of the Porites colony. I think the small Porites colonies are often low in height relative to their diameter, whereas the large Porites colonies are high in height relative to their diameter.

*To our knowledge, no study has demonstrated differences in the colony height/diameter ratio between juveniles and adult massive Porites. These corals typically grow in a hemispherical shape and based on our observations they largely preserve this shape throughout their life. Several studies have looked at the growth of different coral morphotypes, including massive corals* [10,11]*. However, none of these studies reported changes in the shape of massive corals that correlate with colony size.*

*Only in specific circumstances such as very shallow habitats where large massive Porites become exposed to the air in low tide this can result in the death of the top portion of the colony and the subsequent formation of microatolls. In these cases, the live coral surface remains constrained to the sides of the colony and produces a ‘flattened’ colony. We did not observe any of this type of colonies in our study location and therefore, this would have not had an effect on our scar counts.*

The height of the smaller colonies is only a few centimetres at most, so parrotfishes can easily access to the whole surface of small Porites colonies. However, a large Porites colony (Fig. 4 shows that the largest colony is about 70 cm high) might be several tens of centimetres high, could it be that parrotfishes, swimming near the seafloor, only bite the sides of the colony? If so, the density of bite marks will decrease. Additionally, I wonder that the relationship in Fig. 4A is ambiguous, whereas the relationships in Fig. 4B are all too apparent. This gap is particularly large for FLAT.

*Parrotfishes can easily access the entire colony regardless of the size of the colony as demonstrated by the presence of scars throughout the colony surface. Although parrotfishes are reef-associated fishes, they are not bottom-dwellers. They often swim a few feet above the reef substratum. Thus, the top of large Porites is certainly within reach of parrotfishes and many scars were indeed observed on our images of both small and large colonies.*

*It is possible that the apparent lack of a positive relationship between the #scars and the colony size on the reef flat (Fig 4A) is due to A) a smaller range of colony size and B) the comparatively lower number of colonies found on this habitat compared to the other three habitats.*

Is there no result about spatial distribution of Porites in each habitat? Also, the size density distribution of Porites colonies is also helpful. I think these information are important, because the higher the frequency of occurrence, the higher the encounter rate for parrotfishes and the more frequently being bitten. Usually, smaller coral colonies are more frequent than larger colonies. This can be analysed by adding the size frequency distribution of Porites colonies as an explanatory variable. cf. the frequency of each colony can be obtained using the 'density' function in R.

*Thank you for these suggestions. Unfortunately, data on the spatial distribution of massive Porites colonies within each reef habitat is unavailable but we agree with the reviewer that this is an interesting factor that is worth incorporating in future studies.*

*As suggested, we now present the size density distribution of massive Porites colonies for each habitat in Figure S1 which is referenced in the main text. We have incorporated the following text:*

*Lines 207/208 – “Because the size of colonies varied within and across the reef zones (S1 Fig), …”*

The illustration of the habitat types below in Figs. 2, 3, and 4 should be included in Fig.1 rather than showing in every figures. Also, the names of habitat type should be written rather than illustration in Figs. 3 and 4. The photos (Fig. 4c) also should be moved to Fig. 1.

*Thanks for the suggestion. We have moved the illustration of the reef profile to Fig 1 as suggested and we have also removed it from figs 2, 3, and 4. We would like to keep the images in Fig 4c where they are to illustrate differences in size and scar densities.*

The arrows in Fig. 3 should be avoided, since the arrows prevent readers from examining this figure without preconceptions.

*Arrows have been removed as suggested.*

In Table S1, weight of the AIC is usually called as “Akaike weights”, and “WAIC” usually represents Watanabe Akaike Information Criterion, which used in Bayesian model selections. To avoid misleading, different abbreviation should be used. I think “w” or “Wi” (“i” represents ID for each model) is more common for Akaike weights.

*Thanks for pointing this out. We have included the “w” and we now refer to “wAIC” as “Akaike Information Criterion weights”.*

L171–173: Scar density should not be analysed by a GLM with a gamma distribution, since number of scars is a discrete countable variable. GLM with a negative binomial distribution should be used also in this case, but the surface area should be included as an “offset” term, i.e., adding “+ offset(log(SURFACE_AREA))” to the formula of GLM in R.

*We agree with the reviewer and we have corrected the model accordingly. Specifically, we have edited lines 192/194 in the Methods section, and we have updated the summary of the model output in Table S5 (ESM). The correction of this model did not have any impact on the significance of each of the coefficients.*

L174–175, Fig. 3 and Table S2: information theory (AIC model selection) and frequentist (Tukey post-hoc comparisons) should not be mixed in a single analysis. Information theory is also the best choice to perform multiple comparisons among groups, since its statistical power does not decrease with the number of comparisons unlike frequentist post-hoc tests. Just construct models of possible grouping from Slope to Back, and compare them with the AIC.

*Sorry for the lack of clarity. We employed information theory to select between competing model structures and Tukey tests only to run pairwise comparisons of estimates of coefficients (i.e., parrotfish abundance) for each reef zone between 2008 and 2018.*

REFERENCES

1.	Bonaldo RM, Bellwood DR. Parrotfish predation on massive Porites on the Great Barrier Reef. Coral Reefs. 2011;30: 259–269. doi:10.1007/s00338-010-0669-3

2.	Bellwood DR, Choat JH. A functional analysis of grazing in parrotfishes (family Scaridae): the ecological implications. Environ Biol Fishes. 1990;28: 189–214. doi:10.1007/BF00751035

3.	Bellwood DR. The functional morphology, systematics and behavioural ecology of parrotfishes (family Scaridae). James Cook University. 1985.

4.	Bonaldo RM, Hoey AS, Bellwood DR. The ecosystem roles of parrotfishes on tropical reefs. Oceanogr Mar Biol An Annu Rev. 2014;52: 81–132.

5.	Goatley CHR, Bellwood DR. The roles of dimensionality, canopies and complexity in ecosystem monitoring. PLoS One. 2011;6. doi:10.1371/journal.pone.0027307

6.	Burkepile DE. Context-dependent corallivory by parrotfishes in a Caribbean reef ecosystem. Coral Reefs. 2012;31: 111–120. doi:10.1007/s00338-011-0824-5

7.	Bellwood DR, Tebbett SB, Bellwood O, Mihalitsis M, Morais RA, Streit RP, et al. The role of the reef flat in coral reef trophodynamics: Past, present, and future. Ecol Evol. 2018; 1–12. doi:10.1002/ece3.3967

8.	Madin JS, Connolly SR. Ecological consequences of major hydrodynamic disturbances on coral reefs. Nature. 2006;444: 477–480. doi:10.1038/nature05328

9.	Madin JS, Baird AH, Dornelas M, Connolly SR. Mechanical vulnerability explains size-dependent mortality of reef corals. Ecol Lett. 2014;17: 1008–1015. doi:10.1111/ele.12306

10.	Dornelas M, Madin JS, Baird AH, Connolly SR. Allometric growth in reef-building corals. Proc R Soc B Biol Sci. 2017;284. doi:10.1098/rspb.2017.0053

11.	Lough JM, Barnes DJ. Environmental controls on growth of the massive coral Porites. J Exp Mar Bio Ecol. 2000;245: 225–243. doi:10.1016/S0022-0981(99)00168-9
